# Searching for type 2 diabetes prevention interventions in public health and community settings: protocol for a scoping review

**DOI:** 10.1136/bmjopen-2025-109248

**Published:** 2026-01-13

**Authors:** Dominique Michels, Carolin Walter, Alana Grathwohl-Karl, Johanna Pfau, Hannah Haumann, Stefanie Joos, Daniela Fröhlich

**Affiliations:** 1 Institute for General Practice and Interprofessional Care, University Hospital Tübingen, Tübingen, BW, Germany; 2 Institute of Health Sciences, Department of Population-Based Medicine, University Hospital Tübingen, Tübingen, BW, Germany

**Keywords:** Diabetes Mellitus, Type 2, Preventive Health Services, PREVENTIVE MEDICINE

## Abstract

**Abstract:**

**Introduction:**

Type 2 diabetes is a growing global health challenge that requires effective prevention strategies. Public health and community-based approaches play an essential role in reaching vulnerable populations and addressing broader determinants of health. This protocol outlines a scoping review aimed at systematically mapping the existing evidence on lifestyle-based diabetes prevention interventions implemented in public health and community contexts.

**Methods and analysis:**

A systematic literature search will be conducted to identify relevant studies published in English or German from 1 January 2014 onwards. The following databases will be searched: PubMed, Web of Science Core Collection, CINAHL (via EBSCO), the Cochrane Central Register of Controlled Trials, the Cochrane Database of Systematic Reviews (via OVID) and ClinicalTrials.gov. Relevant websites and grey literature sources will be searched to identify further eligible studies. (Cluster-)randomised controlled trials, non-randomised controlled trials and clinical trials will be included. These must examine nutrition-based, physical activity-based or lifestyle-based interventions aimed at preventing type 2 diabetes in healthy adults or individuals with pre-diabetes, implemented in public health or community settings. Case reports and studies involving medical therapies or pharmacological interventions will be excluded. The literature search started in May 2025 and is expected to be completed by the end of December 2025.

**Ethics and dissemination:**

As this scoping review is based on the secondary analysis of publicly available data, no ethical approval is required. Our dissemination strategy includes publication in peer-reviewed journals, presentations at academic conferences and targeted dissemination to relevant interest holders.

**Study registration:**

This project has been registered at Open Science Framework (https://osf.io/zafg5/), as PROSPERO does not accept registrations for scoping reviews.

**STRENGTHS AND LIMITATIONS OF THIS STUDY**

- The protocol follows Joanna Briggs Institute and Preferred Reporting Items for Systematic Reviews and Meta-Analyses Extension for Scoping Reviews guidance, ensuring internationally recognised methodological standards.
- A comprehensive and systematic search strategy will be implemented across multiple electronic databases and grey literature sources, ensuring broad coverage of relevant evidence.
- Limiting the inclusion criteria to studies published in English and German, as well as to specific study types, may lead to (language) bias and potentially exclude relevant research conducted in other languages or using other types of studies.
- Critical appraisal is intentionally not carried out, in line with scoping review methodology.

## Introduction

### Rationale

 Type 2 diabetes mellitus (T2DM) is one of the most significant public health challenges globally, with increasing prevalence rates projected to affect over 853 million adults by 2050.^1^ This chronic condition is associated with substantial health and economic burdens, particularly due to its long-term complications and comorbidities.

Lifestyle-based interventions, defined as structured programmes or strategies aimed at promoting healthier behaviours through changes in nutrition, physical activity or broader lifestyle habits, have demonstrated effectiveness in delaying or preventing the onset of T2DM among individuals with pre-diabetes or elevated metabolic risk.^2 3^ Over the past two decades, several national and regional initiatives have been launched with the aim of translating evidence-based approaches into practical and scalable prevention strategies.^4^,^7^

Findings from countries such as Finland, the USA and the UK demonstrate that substantial health benefits can be achieved under real-world conditions.^8^,^10^ For example, the Finnish National Diabetes Prevention Programme reported a risk reduction of T2DM of 69% after a 1-year intervention in individuals with high risk of T2DM who were able to lose 5% of their weight.^10^ However, these interventions often experience limited reach, participation and adherence among specific population groups. Young adults, racial and ethnic minorities and socially disadvantaged individuals are frequently under-represented in existing programmes.^8^,^10^

Furthermore, the implementation of such interventions in real-world settings has proven challenging due to structural and operational issues, including limited financial and human resources, inadequate institutional integration and heterogeneity of the target population.^6 8 11^

Considering these challenges, public health and community-based settings are gaining increasing attention in public health prevention efforts.^12 13^ Due to its close ties to the local population, these settings enable the development and implementation of diabetes prevention programmes tailored to specific needs. This may increase acceptance and participation, thereby contributing to the long-term success of diabetes prevention programmes.

### Objectives

The objective of this review is to systematically identify and examine diabetes prevention interventions that (a) target healthy individuals or those with pre-diabetes (defined by the WHO and American Diabetes Association (ADA)^14 15^), (b) focus on lifestyle change and/or change in behaviour and (c) are implemented in public health and community settings. By examining the design, target populations, implementation approaches and reported outcomes of such interventions, this review will provide an overview of the current practices in the field. It also aims to highlight promising approaches and identify gaps in the evidence base that warrant further research or policy attention.

Previous reviews have primarily focused on diabetes prevention interventions targeting communities in the sense of populations living in a specific region.^12 13^ They did not address interventions implemented within public health or community settings (eg, public health departments, municipal/community centres), thereby leaving a gap that the present study aims to fill.

The review seeks to answer the following questions:

- How are diabetes prevention interventions in public health or the community setting conceptualised, designed, implemented and evaluated?
- Which target populations are addressed by diabetes prevention interventions implemented in public health or community settings?
- What gaps exist in the current landscape of diabetes prevention interventions in public health and community contexts?

## Methods and analysis

### Study design

Given the broad nature of the research question and the anticipated variability in both the type and quality of available evidence, a scoping review is considered the most appropriate approach. This method enables a thorough overview of the existing literature while helping to identify gaps in current knowledge and research. The proposed scoping review will be conducted in accordance with the Joanna Briggs Institute methodology for scoping reviews.^16 17^

Any modifications to the Open Science Framework registration or to this protocol will be documented along with a rationale for the change.

### Conceptual framework

To conceptualise our research question and define the selection criteria, we applied the population, concept and context (PCC) framework based on the following components:

- Population: we will focus on healthy adults (18 years and older), as well as adults at increased risk of T2DM, such as those with pre-diabetes or metabolic syndrome.
- Concept: we will include lifestyle-based interventions aimed at preventing T2DM, with a focus on components such as nutrition, physical activity and general lifestyle behaviours.
- Context: our review will consider interventions implemented in public health or community settings (eg, public health departments, municipal/community health centres, the Centers for Disease Control and Prevention (CDC), primary healthcare facilities, local health services, health planning organisations).

In collaboration with a medical librarian, a search string based on the PCC framework will be developed and adapted to various databases and platforms. The full search strings for four main databases will be available in the online supplemental material.

### Inclusion and exclusion criteria

We will include experimental and quasi-experimental study designs, such as (cluster-)randomised controlled trials, non-randomised controlled trials and clinical trials. Studies identified in review articles will be included where considered relevant. Case reports and articles published in languages other than English or German will be excluded. Furthermore, studies involving medical therapies or pharmacological interventions will not be considered. The review will not be limited to any cultural, racial, gender or geographical context.

To ensure relevance to current public health practice, the present review is limited to studies published from 2014 onwards, capturing developments over the past decade in intervention design, implementation strategies and policy context.

### Literature search

We will conduct a systematic search to identify relevant studies from both published and unpublished sources. The electronic databases to be searched include PubMed, Web of Science Core Collection, CINAHL (EBSCO), the Cochrane Central Register of Controlled Trials, the Cochrane Database of Systematic Reviews via OVID and ClinicalTrials.gov.

After completing the database searches, we will manually review the websites of key organisations, including the WHO, the CDC, the International Diabetes Federation and the ADA.

In addition, we will conduct a structured search of Google Scholar, with relevance determined based on the first five pages of results. To capture grey literature, we will search ProQuest and OAIster. Furthermore, we will screen the reference lists of all included studies to identify any additional relevant sources.

### Study selection and screening

Following the search, we will compile all identified citations and upload them into EndNote 21 (Clarivate Analytics, Philadelphia, USA) to remove duplicates. The deduplicated records will then be imported into Covidence (Veritas Health Innovation, Melbourne, Australia) for screening.

After conducting a pilot screening, two reviewers will independently assess titles and abstracts to determine eligibility based on the predefined inclusion criteria. Studies deemed potentially relevant will undergo full-text review, again by two independent reviewers.

For each full-text article excluded, we will document the reason for exclusion. Any disagreements during the screening process will be resolved through discussion. If consensus cannot be reached, a third reviewer will be consulted.

We will report the results of the search and the study selection process in full in the final scoping review and present them using a Preferred Reporting Items for Systematic Reviews and Meta-Analyses extension for Scoping Reviews flow diagram.^18^

### Data extraction

We will extract data from all studies included in the scoping review using a data extraction form developed by the review team. This extraction form is designed to capture both general study characteristics and specific information relevant to our research objectives. The data to be extracted will include the following: (1) bibliographic information (eg, authors, year of publication, country); (2) study design; (3) population characteristics (eg, recruitment process, inclusion/exclusion criteria, age, gender, number of participants); (4) intervention details and contextual factors (eg, curriculum of intervention/control group, duration, location/setting, community/stakeholder involvement, theoretical framework); (5) study objectives; (6) outcome measurements (eg, time of assessment/follow-ups, assessment tools, primary and secondary outcomes); and (7) key findings relevant to the review questions.

Two reviewers will independently perform the initial data extraction from the included studies, and a third reviewer will cross-check the data to ensure accuracy and consistency. Discrepancies during data extraction will be resolved through discussion or, if necessary, in consultation with another reviewer. If needed, we will contact study authors to clarify or obtain missing information.

We will pilot and iteratively refine the data extraction tool throughout the review process to ensure its completeness and relevance. All modifications made to the extraction form will be documented and reported in the final scoping review.

### Data synthesis

Due to the nature of a scoping review, we will not perform a formal critical appraisal of the included studies, as the primary aim is to map the available evidence rather than to assess methodological quality.

We will provide a systematic narrative synthesis, presenting information in both text and tabular formats to summarise and explain the characteristics and findings of the included studies. The narrative synthesis will explore relationships and findings within and between the included studies.

### Research project

The planned scoping review is part of the overarching project ‘From research to practice: promoting diabetes prevention in public health services through participatory collaboration’. The literature search started in May 2025 and is expected to be completed by the end of December 2025. As Work Package 2, the scoping review directly follows Work Package 1, which involved semistructured interviews with representatives of the public health sector in Baden-Württemberg (Germany) to identify current practices and needs. Findings from Work Packages 1 and 2 will be synthesised and subsequently discussed with public health stakeholders in Baden-Württemberg during a participatory workshop as part of the final Work Package 3.

### Patient and public involvement

As this scoping review will use secondary data sources, neither patients nor the public will be involved in its design, conduct or reporting.

### Ethics and dissemination

This project is part of a research initiative focusing on diabetes prevention in the public health and community context. The overarching research initiative has obtained ethical approval (206/2024BO2) from the University of Tuebingen. As this protocol reports a scoping review that involves secondary analysis of previously collected data, it does not require any additional ethical approval.

Our dissemination strategy includes publication in peer-reviewed journals, presentations at academic conferences and targeted dissemination to relevant interest holders.

## Supplementary material

10.1136/bmjopen-2025-109248 online supplemental file 1
