# Supplementary material for: Searching for type 2 diabetes prevention interventions in public health and community settings: protocol for a scoping review
Source: BMJ Open. 2026 Jan 13;16(1):e109248. doi: 10.1136/bmjopen-2025-109248 (PMC12815110; doi:10.1136/bmjopen-2025-109248)
Supplement: Supplementary file 2 [file bmjopen-2025-109248.reviewer_comments.pdf]

## PEER REVIEW HISTORY

BMJ Open publishes all reviews undertaken for accepted manuscripts. Reviewers are asked to complete a checklist review form and are provided with free text boxes to elaborate on their assessment. These free text comments are reproduced below.

### ARTICLE DETAILS

**Title (Provisional)**

Searching For Type 2 Diabetes Prevention Interventions In Public Health And Community Settings: Protocol For A Scoping Review

**Authors**

Michels, Dominique; Walter, Carolin; Grathwohl-Karl, Alana; Pfau, Johanna; Haumann, Hannah; Joos, Stefanie; Fröhlich, Daniela

---

### VERSION 1 - REVIEW

---

| | |
|---|---|
| **Reviewer** | **1** |
| **Name** | **Doherty, Anne** |
| **Affiliation** | **University College Dublin, Psychiatry** |
| **Date** | **16-Sep-2025** |
| **COI** | **None** |

---

Many thanks for requesting peer review of this interesting paper. It is a protocol paper for a planned scoping review which aims to examine interventions in community and public health settings which aim to reduce the overall incidence of type 2 diabetes mellitus in those with prediabetes or without diabetes.

The planned methodology is appropriate to the research question, and the paper is well written.

Minor points:

1. Given that the focus of this work is T2DM, it might be good to specify this in the title.

2. How will this study deal with high risk populations within community populations, for example people with mental illness (in particular psychosis)?

---

| | |
|---|---|
| **Reviewer** | **2** |
| **Name** | **Manyara, Anthony Muchai** |
| **Affiliation** | **University of Bristol** |
| **Date** | **06-Oct-2025** |

| | |
|---|---|
| **COI** | **None** |

This protocol presents a scoping review on public and community based interventions for diabetes prevention. Given the increasing burden of diabetes and importance of prevention, this is a timely and relevant topic. However, I have some concerns about the protocol as it is

There is no summary of existing reviews on this topic to identify a gap that this review is aiming to fill

The scope of the review also seems quite broad to be feasibly possible given that this a project with other work packages and perhaps narrowing down to a particular area based on gaps of existing reviews would be the way to go

Mention of work packages come at the end and there is no mention of this earlier in the manuscript

It is common to use PRISMA Scoping Reviews extension for reporting scoping reviews and their protocols

Search for public health interventions maybe better done in city council and public health department websites for evaluation reports rather organisations such as WHO. Again what countries would this focus on as they are quite many globally.

## VERSION 1 - AUTHOR RESPONSE

| Reviewer: 1<br>Dr. Anne Doherty, University College Dublin, Mater Misericordiae University Hospital | |
|---|---|
| Given that the focus of this work is T2DM, it might be good to specify this in the title. | The title has now been changed to: 'Searching for Type 2 Diabetes Prevention Interventions...' (line 1, p.1) |
| How will this study deal with high risk populations within community populations, for example people with mental illness (in particular psychosis)? | As this is a scoping review, the initial focus is on identifying and mapping existing literature. If the studies identified describe interventions for specific target groups, such as individuals experiencing mental health issues including psychosis, these will be reported, and considered in the discussion. |
| Reviewer: 2<br>Dr. Anthony Muchai Manyara, University of Bristol | |
| There is no summary of existing reviews on this topic to identify a gap that this review is aiming to fill | To date, there are no reviews that specifically address interventions carried out in the field of public health and in municipal institutions (e.g., health departments, ...). To highlight this point more clearly, a paragraph in lines 129-133, p.4 was added under "objectives" and two review sources were added. (line 130, p.4) |
| The scope of the review also seems quite broad to be feasibly possible given that this a project with other work packages and perhaps narrowing down to a particular area based on gaps of existing reviews would be the way to go | As a scoping review, the aim of this study is intentionally broad, as it focuses on mapping the available evidence rather than answering a narrowly defined question, which distinguishes it from other types of reviews. Narrowing the scope to a specific area could risk excluding important findings from other countries. Given that our review is limited to database searches, we consider the current scope feasible within the framework of the project and its other work packages. |
| Mention of work packages come at the end and there is no mention of this earlier in the manuscript | Mentions of the work packages have now been incorporated earlier in the manuscript under a dedicated subchapter titled "Research Project," to provide clearer context. (lines 240-249, p.7) |
| It is common to use PRISMA Scoping Reviews extension for reporting scoping reviews and their protocols | The PRISMA-ScR checklist was provided with the initial submission and is also referenced in the manuscript (see Section 2.5, Study Selection and Screening). |
| Search for public health interventions maybe better done in city council and public health department websites for evaluation reports rather organisations such as WHO. Again what countries would this focus on as they are quite many globally. | In Work Package 1, a pilot search for interventions on the city council and public health department level in the federal state Baden-Württemberg in Germany was conducted. Based on this experience, conducting such searches internationally proved to be largely unfeasible. Therefore, for |

| | |
|---|---|
| | this scoping review, we have limited our search to database sources only. |

## VERSION 2 - REVIEW

| | |
|---|---|
| **Reviewer** | **1** |
| **Name** | **Doherty, Anne** |
| **Affiliation** | **University College Dublin, Psychiatry** |
| **Date** | **07-Dec-2025** |
| **COI** | |

The previous (very minor) concerns have been addressed. I have no further comments
